# Determinants in early life for asthma development

**DOI:** 10.1186/1710-1492-5-6

**Published:** 2009-11-09

**Authors:** Hugo PS Van Bever

**Affiliations:** 1 Department of Pediatrics, National University Singapore, Singapore, 119260, Singapore

## Abstract

A reliable screening test in newborns for the subsequent development of bronchial asthma (BA) has not been found yet. This is mainly due to the complexity of BA, being made up by different types and underlying mechanisms. In different studies, a number of risk factors for BA have been identified. These include a positive family history of BA, passive smoking (also during pregnancy), prematurity (including pulmonary infections, RDS and BPD), early viral respiratory infections (such as RSV-bronchiolitis), male gender, early lung function abnormalities and atopic constitution. The major risk factor for persistent BA is an underlying allergic constitution. Therefore, early symptoms and markers of allergy (i.e. The Allergic March) and a positive family history for allergy should be considered as important risk factors for the development of BA.

## Background

### What is asthma?

Bronchial asthma (BA) is more than just one disease of the lower airways, and is now considered to be a syndrome, the asthma syndrome, made up by a spectrum of different conditions that are manifested by recurrent symptoms of bronchial obstruction, i.e. recurrent symptoms of wheezing and/or cough, and having as major feature the existence of bronchial hyperreactivity, as a consequence of chronic bronchial inflammation. A number of classifications of BA have been proposed, based on severity, etiology or age of the patient. In one classification two major subtypes of BA are distinguished: primary and secondary BA. Furthermore, it is recognized that BA can also be a consequence of an underlying specific airway disease (Appendix 1) [1].

Primary asthma can be considered as a type of asthma of which the etiology can be situated in the bronchi itself, i.e. bronchial hyperresponsiveness to a number of triggers, such as allergens, viruses and pollution. Secondary asthma is a type of asthma of which the etiology is situated outside the bronchi, such as in asthma maintained by chronic rhino-sinusitis or gastro-oesophageal reflux. In this latter type, bronchial hyperresponsiveness is a secondary phenomenon, indicating that treatment should be focused on pathologies outside the lower airways. Asthma with specific airway diseases is the existence of asthmatic symptoms (i.e. recurrent wheeze and/or cough) in specific airway diseases such as cystic fibrosis, structural bronchial malformations, immune deficiencies, ciliar dyskinesia and others.

This classification of BA not only refers to the different causes of asthma, but also to a different prognosis, suggesting that specific treatment regimens should be used. This is clearly illustrated by looking at asthma in children younger than 3 years of age. The evidence suggests that recurrent obstructive symptoms (i.e. recurrent wheezing) remit in a large number of these children who develop these symptoms during the first 3 years of life. In these children, recurrent wheezing is usually evoked by viral infections and low lung function parameters seem to be the main risk factor for these transient episodes. On the other hand, children who will go on to develop persistent wheezing beyond infancy and early childhood usually have a family history of asthma and allergies and present with allergic symptoms very early in life [2,3].

### Determinants in early life for asthma development

Since asthma (i.e. recurrent wheezing) constitutes different types, it is very difficult to predict its occurrence, especially in newborns. Furthermore, a severe lower airway infection with RSV or with another respiratory virus is able to induce BA in a previously complete healthy baby with a complete negative family history for BA or allergy [4]. Therefore, there are few truly justified recommendations for the prevention of asthma [5]. The GINA guidelines (2006, chapter 4) mention: "... few measures can be recommended for prevention of asthma because the development of the disease is complex and incompletely understood."

However, in different studies, a number of risk factors for BA have been described [1]. These include: 1. a positive family history of BA, 2. passive smoking (prenatally and postnatally), 3. prematurity (including pulmonary infections, RDS and BPD), 4. early viral respiratory infections (such as RSV-bronchiolitis), 5. male gender, 6. early lung function abnormalities and 7. an atopic constitution (Appendix 2).

#### 1. Family history of BA

In a large number of studies it was demonstrated that a positive family history for BA and for atopy (see below) are important risk factors for BA. In a recent study from the South Bronx is was shown that the most important risk factors for BA are Hispanic ethnicity, family history of asthma, and exposure to tobacco smoke [6]. In other studies the effect of early-life environmental exposures on genetic factors has been shown. In a study by Kuiper et al, a modification of the effect of family history of BA on respiratory morbidity by environmental exposures in early life was demonstrated. Postnatal parental smoking and high indoor dust mite allergen levels accentuated the increased risk of wheeze associated with a positive family history, whereas breast-feeding attenuated the increased risk of upper airway pathologies [7].

#### 2. Passive smoking

Although passive exposure to cigarette smoke in young children is a risk factor for respiratory symptoms, childhood asthma, airway hyperresponsiveness and diminished pulmonary function status, no definitive study has implicated passive smoking as a risk factor for the persistence of recurrent wheezing [1]. On the other hand, it seems very acceptable that passive smoking worsens prognosis of BA in young children, based on the observation that lung growth is diminished in children from smoking pregnant women [8].

#### 3. Prematurity

Prematurity with respiratory morbidity, such as RDS, can result in long-term lung damage (bronchopulmonary dysplasia) and bronchial hyperreactivity, which is a predisposing situation for severe viral-induced wheezing during years [9].

#### 4. Viral respiratory infections

RSV lower respiratory tract illnesses in early life are an independent risk factor for the subsequent development of wheezing up to age 11 years. Severe RSV infections, requiring hospitalization, can induce persistent IgE-mediated hypersensitivity reactions up to the age of 7 years [10,11]. The exact mechanisms are fairly unknown, but a RSV-induced switch from Th1 to Th2 features has been shown [12]. However, the relation between RSV infection and subsequent BA is still very much debated. It seems that pre-existing atopy may be a marker for more severe bronchiolitis, and atopy itself predisposes to BA [13].

#### 5. Male gender

Male gender has been demonstrated to be a risk factor for BA in children before the age of 14 years, while female gender to be a risk factor for asthma in adults. In one study it was shown that boys had a higher incidence rate of BA, while girls had a greater deficit in pulmonary function, suggesting a worse long term prognosis in female patients [14]. An explanation for this could be that boys have a higher prevalence of allergic sensitization than girls, while in adults the gender difference is reversed [15].

#### 6. Early lung function abnormalities

Early lung function abnormalities have been associated with an increased risk of recurrent wheezing. In a recent study it was found that poor airway function shortly after birth should be recognized as a risk factor for airflow obstruction in young adults and that prevention of chronic obstructive pulmonary disease might need to start in fetal life [16].

#### 7. Allergy as a major risk factor to develop persistent asthma

The causes of allergy are multi-factorial, and the development of an allergic disease is the result of complex interactions between genetic constitution and environmental factors. Genetic constitution is important, as it is in genetically predisposed individuals that the environment is able to trigger symptoms of allergy. At birth allergic symptoms usually are not present, although it was demonstrated that allergic immune responses already can start during fetal life and that the fetus is able to respond to allergens from week 20 of pregnancy [17]. In young children, eczema and food allergy (diarrhea, vomiting, failure to thrive) are usually the first manifestations of allergy, while in older subjects allergy manifests itself more often as chronic or recurrent asthma and/or allergic rhinitis. This phenomenon of switching from one expression of allergy to another is called the 'Allergic March'.

Among risk factors to develop BA, from a substantial number of studies it was concluded that atopy is one of the most important risk factors [18]. Early allergen exposure seems to be a major trigger, but attempts at prevention by allergen avoidance have produced conflicting results [19]. Moreover, from recent studies it seems that there is no linear relationship between early allergen contacts and the development of BA, as both exposure to high doses and low doses of allergens might have a protective effect, suggesting the existence of a bell-shaped relationship [20].

It is generally accepted that atopy is associated with a poorer prognosis of asthma during childhood [1]. Atopy was associated with persistent wheezing in a cohort of babies at high risk for allergic diseases and was associated with an increased risk for both early and later childhood onset of wheezing [21]. In a follow-up of a 1958 birth cohort, subjects who had asthma or wheezy bronchitis by age 16 years were twice as likely to have a report of wheezing during the preceding year if they had hay fever, allergic rhinitis, or eczema [22]. Furthermore, children experiencing persistent asthma beyond early life have increased serum IgE levels during the first year of life and are more likely than other children to be sensitized to foods [23,24]. In one study a clinical index, based on family history and atopic features, was proposed (Table 1) [25]. In that study it was found that 95% of young wheezy children with a negative index never developed asthma between the ages 6 - 13 years. In another study from Finland, food allergy during the first three years of life was also a risk factor to develop persistence of wheezing until school age [26].

**Table 1:** A clinical index to define asthma risk (from Castro-Rodriguez et al, 2000)

Major Criteria	Minor Criteria
1. Parental asthma*	1. allergic rhinitis*
2. eczema*	2. Wheezing apart from colds
	3. Eosinophilia (> 4%)

*Physician diagnosis of asthma, eczema or allergic rhinitis.

Taken together, it is clear that allergy is a risk factor to develop persistent asthma in infants and young children. Once asthma has itself established in the child, allergy appears not to be an independent determinant of prognosis into adulthood, suggesting that inflammatory processes in the airways run their own courses irrespective of the subject's atopic status [1].

### Determinants in early life of atopy

Early prevention of allergic diseases, including BA, has been regarded as an important corner stone in the management of atopic diseases. Therefore, the identification of reliable screening markers detecting individuals (newborns) at risk has been an area of intense research during the past thirty years. Many efforts have been made to find reliable predictors of atopy which might identify children at risk and allow the initiation of primary preventive strategies at an early stage. As a consequence, various studies have been performed in which markers of atopy in cord blood were assessed [18]. These include genetic markers of allergy, IgE levels, levels of soluble mediators of atopy (cytokines, receptors), determination of receptors connected to bacterial immune defense (linked to the so-called 'Hygiene Hypothesis'), determination of polyunsaturated fatty acids, cytokine profiles of mononuclear cells and markers of antigen presenting cells.

From a number of studies it seems that interferon-gamma (IFN-γ) might be one of the appropriate candidate-markers for the prediction of BA and allergy. Production of IFN-γ has been used as a potential marker for the postnatal immune maturation processes that are associated with the subsequent risk for development of BA or allergic diseases. Studies on cord blood mononuclear cells have shown that subjects who will develop allergic symptoms have a characteristic pattern of response that includes decreased production of IFN-γ, suggesting a Th_2_-type predominance [27,28]. Stern et al found that low IFN-γ production by mitogen-stimulated mononuclear cells at the age of 9 months was associated with an increased risk of wheezing between 2 and 13 years [29]. Guerra et al reported that low IFN-γ production at 3 months of age was associated with recurrent wheeze in the first year of life [30].

Björksten et al. showed that interleukin-4 (IL-4) production by peripheral blood mononuclear cells in early life may be predictive of the subsequent development of allergic symptoms [31]. In another cross-sectional study, no major differences indendritic cell features were found between children from allergic and non-allergic studies. However, no follow-up for wheezing was performed [32]. In a more recent study from Germany, a strong interaction of cord blood adiponectin and history of atopic disease in the mother with respect to the risk of physician-reported asthma or obstructive bronchitis was found (p = 0.006). The authors concluded that in children of mothers with a history of atopy, concentrations of adiponectin in cord blood could play an important role in determining risk of wheezing disorders in early childhood [33].

Although the findings of these studies have improved current knowledge on the initial mechanisms and evolution of atopy (e.g. the prenatal events of atopy), most of these parameters that were studied did not show any reliable association or predictive value, and studies showed conflicting results. The main reasons for screening difficulties in atopic diseases include:

1/allergic manifestations are usually not present at birth, but usually start during the first years of life, as a consequence of interactions between genetic constitution and environment.

2/features of allergy can be present in healthy persons (e.g. positive skin prick tests were found in > 10% of healthy children).

3/so-called symptoms of allergy (asthma, rhinitis, eczema) can be present without the presence of allergy (= patients have negative skin prick tests).

4/allergy is multi-factorial (a large number of genes involved in allergy have been described), dynamic, unpredictable, and certainly not a constant disease.

Nowadays we still have no reliable predictive marker(s) of allergy, although, in theory, because of its large burden of allergic diseases to society, it would be of value to identify newborns at risk. Furthermore, the effectiveness of specific primary preventive measures is very limited for the newborn at risk (apart from breast feeding and avoidance of passive smoking). Nowadays, the best screening for allergy still is an extensive family history (including questions on childhood of the parents), in combination with an objective assessment of allergy in the parents or siblings using skin prick testing or determination of specific serum IgE.

## Conclusion

A reliable screening test in newborns for the subsequent development of BA has not been found yet. This is mainly due to the complexity of BA, which is made up by different types and underlying mechanisms (i.e. The Asthma Syndrome). However, in different studies, a number of risk factors for BA have been identified, such as: a positive family history of BA, passive smoking (also during pregnancy), prematurity (including pulmonary infections, RDS and BPD), early viral respiratory infections (such as RSV-bronchiolitis), male gender, early lung function abnormalities and atopic constitution. The major risk factor for persistent BA is an underlying allergic constitution. Early symptoms and markers of allergy (i.e. The Allergic March) and a positive family history for allergy should be considered as important risk factors for the development of asthma. As such, the profile of the newborn at risk to develop BA can be summarized as follows: it is a male, prematurely born infant whose parents suffer from asthma and/or allergy and who smoke. The baby has a dry skin with eczematous patches and develops a severe bronchiolitis early in life for which he had to be admitted to PICU for 1 week.

## Competing interests

The author declares that they have no competing interests.

## Appendix 1 - Different types of childhood asthma: 'The Asthma Syndrome'

-PRIMARY ASTHMA

- Viral-induced asthma

- Allergic asthma

- Non-specific asthma (pollution, exercise)

-SECONDARY ASTHMA

- Asthma associated to upper airway pathology (rhino-sinusitis, adenoiditis)

- Asthma associated to gastro-oesophageal reflux

-ASTHMA WITH SPECIFIC AIRWAY DISEASES

## Appendix 2 - Risk factors in newborns for developing BA

1. Family history of BA

2. Passive smoking

3. Prematurity

4. Early viral airway infection

5. Male gender

6. Early lung function abnormalities

7. Atopic constitution
